# Yeast secreted protein PgSCP interacts with citrus transcription factors CsFAR1 to enhance green mold resistance in fruit

**DOI:** 10.1093/hr/uhaf339

**Published:** 2025-12-08

**Authors:** Ou Chen, Rui Huang, Yao Xu, Shixiang Yao, Jian Ming, Kaifang Zeng

**Affiliations:** College of Food Science, Southwest University, Chongqing 400715, China; College of Food Science, Southwest University, Chongqing 400715, China; College of Food Science, Southwest University, Chongqing 400715, China; College of Food Science, Southwest University, Chongqing 400715, China; Research Center for Fruit and Vegetable Logistics Preservation and Nutritional Quality Control, Southwest University, Chongqing 400715, China; College of Food Science, Southwest University, Chongqing 400715, China; Research Center for Fruit and Vegetable Logistics Preservation and Nutritional Quality Control, Southwest University, Chongqing 400715, China; College of Food Science, Southwest University, Chongqing 400715, China; National Citrus Engineering Research Center, Southwest University, Chongqing 400712, China; Research Center for Fruit and Vegetable Logistics Preservation and Nutritional Quality Control, Southwest University, Chongqing 400715, China

## Abstract

Green mold caused by *Penicillium digitatum* significantly impacts the citrus industry economically. Enhancing postharvest disease resistance in citrus fruit remains challenging due to the complex pathogen-citrus interaction. Previous researches have indicated that PgSCP, a cysteine-rich secretory protein derived from *Pichia galeiformis*, activates resistance responses in citrus fruit. However, the precise molecular mechanisms underlying this effect remain unclear. This study showed that PgSCP enhances disease resistance gene expression and substance accumulation in citrus fruit. Additionally, potential citrus proteins that may interact with PgSCP was identified. Among these, four candidate transcription factors were identified: CsFAR1, CsMIKC, CsLBD, and CsGRAS. Subsequent validation demonstrated that PgSCP interacts with the citrus transcription factor CsFAR1. Transient overexpression analysis demonstrated that CsFAR1 positively regulates resistance to green mold, and CsFAR1 also enhances the disease resistance gene expression in citrus fruit. The CsFAR1 protein enhances resistance by activating *DHAPS-1*, *GSH1*, *ACO1*, *INVA, PAL6*, *OMT*, *CYP73A16*, *CCOAOMT1*, *CYP73A4*, and *PER16*. These findings suggest that the yeast-secreted protein PgSCP may act as an elicitor that interacts with citrus transcription factors CsFAR1 to enhance host defense responses, thereby contributing to improved postharvest resistance to green mold.

## Introduction

Citrus fruit represent one of the most widely cultivated fruit crops worldwide, ranking first in both cultivation area and production volume. However, citrus fruit exhibit a high susceptibility to pathogen infection during postharvest storage and transportation, resulting in decay and degradation that contribute to substantial economic losses [1]. Consequently, it is imperative to implement effective strategies for the prevention and management of postharvest diseases. The prevalent use of chemical fungicides in the industry has been associated with the rapid development of pathogen resistance, alongside adverse impacts on environmental and human health. Thus, there is a critical need to explore and adopt sustainable and environmentally benign disease control technologies [2]. Among these, biocontrol yeasts have emerged as promising alternatives due to their genetic stability, broad ecological adaptability, low nutritional requirements, and strong antagonistic activity against a range of pathogens. The application of biocontrol yeasts for the biological control of postharvest diseases is regarded as one of the most promising alternatives to fungicides within the domain of postharvest management [3].

One of the main mechanisms by which yeast controls postharvest diseases in fruits is by inducing the fruits to strengthen disease resistance [4]. Recent research has shown that microorganisms can secrete small proteins capable of modulating host defense responses. For instance, *Verticillium dahliae* secretes effector proteins that enhance plant immunity [5]. *Pichia galeiformis* has been reported to enhance disease resistance in citrus fruit through its secreted proteins, among which PgSCP (*P. galeiformis* Secreted Cysteine-rich Protein; gene 0649) plays a pivotal role in this protective process [6]. PgSCP is a secreted cysteine-rich protein produced by *P. galeiformis*. However, the precise mechanism through which PgSCP modulates fruit resistance to green mold remains inadequately understood. In particular, the manner in which PgSCP interacts with biotic factors, such as transcription factors within the fruit, to affect the postharvest disease resistance response warrants further investigation. Considering that transcription factors serve as central regulators of plant immunity, it is plausible that PgSCP may influence citrus resistance by affecting transcriptional networks involved in downstream defense pathways.

Transcription factors, alternatively referred to as trans-acting factors, constitute a category of proteins that modulate gene transcription through their interaction with specific DNA sequences within the genome. A multitude of transcription factors have been documented to engage in defense mechanisms against diseases and stressors in postharvest fruits by orchestrating the expression of downstream genes associated with disease resistance. For example, members of the WRKY and NAC transcription factor families directly bind to the promoters of genes involved in plant disease, thereby activating their expression and enhancing plant resistance to pathogens [7].

Microbially secreted proteins can influence plant defense by interacting with host transcription factors, thereby modulating their regulatory activity and downstream defense signaling. For example, NAC transcription factors have been shown to interact with pathogen-derived effectors, leading to altered transcription of defense-related genes and changes in plant resistance [8]. In plants, FAR1 transcription factors are known to participate in stress responses and may also contribute to disease resistance regulation. To identify plant proteins that potentially interact with microbial secreted proteins, pull-down assays coupled with mass spectrometry are widely used. This combined approach enables efficient identification and characterization of interacting partners. For instance, immunoprecipitation and mass spectrometry analyses were employed to reveal the interaction between the secreted protein CRN78 from *Phytophthora sojae* and the aquaporin NbPIP2;2 [9]. Similarly, the potato late blight pathogen *Phytophthora infestans* secretes the effector protein Pi03192, which interacts with NAC transcription factors, impeding their nuclear localization and consequently inhibiting the resistance of potatoes to this pathogenic oomycete [10]. The rust pathogen *Melampsora larici-populina* secretes the effector protein Mlp124478, which localizes to the host cell nucleus and interacts with the transcription factor TGA1a. This interaction inhibits the expression of downstream defense-related genes, thereby suppressing the defense response of poplar against rust disease [11].

Effector proteins secreted by pathogenic microorganisms are known to interfere with host immune signaling by targeting transcription factors within plant cells. For instance, the secreted protein PstGSRE1 from the wheat stripe rust fungus interacts with the reactive oxygen species (ROS)-related transcription factor TaLOL2, resulting in the inhibition of the TaLOL2-mediated ROS response and consequently suppressing the defense mechanisms in wheat [12]. The bacterial wilt pathogen *Ralstonia solanacearum* secretes the effector protein RipAB, which localizes to the nucleus of *Nicotiana benthamiana* cells, where it interacts with the TGA transcription factor. This interaction disrupts the salicylic acid signaling pathway and subsequently inhibits the disease resistance responses in both *Arabidopsis* and tomato plants [13].

Studies have further demonstrated that cysteine-rich secretory proteins from pathogens have also been reported to interact with transcription factors to modulate host immunity. For example, the cysteine-rich secretory protein PpEC23, secreted by the soybean rust pathogen *Phakopsora pachyrhizi*, has been shown to interact with the soybean immune negative regulatory transcription factor GmSPL12l, thereby inhibiting the soybean defense response [14]. Likewise, the cysteine-rich secretory protein VdSCP41 secreted by *V. dahliae* interacts with the cotton transcription factor GhCBP60b and inhibits its transcriptional activity, thereby suppressing the cotton’s resistance to *V. dahliae* [15]. In contrast, the cysteine-rich secreted protein ScSCP of *Sclerotinia sclerotiorum* has been shown to activate immune responses in *Arabidopsis thaliana*, *N. benthamiana*, and *Solanum lycopersicum*. Exogenous application of ScSCP induces broad-spectrum resistance against bacterial and fungal pathogens in plants [16] . So, it’s possible that non-pathogenic or beneficial microbes may secrete SCP-like proteins that trigger defense activation.

Transcription factors play pivotal roles in mediating the disease resistance of postharvest fruits. *FaWRKY11* overexpression in strawberry upregulates resistance- and hormone-related genes, thereby improving defense against *Botrytis cinerea* [17]. The ethylene-responsive factor FaERF2 also contributes to resistance by activating the β-1,3-glucanase gene [18]. Similarly, overexpression of *CsMYB96* in citrus fruit enhances the biosynthesis of salicylic acid (SA) and phenolic compounds, strengthening resistance against *Penicillium italicum* [19]. CsWRKY70 facilitates the accumulation of methyl salicylate in citrus fruit through the activation of *CsSAMT* expression, thereby inhibiting the incidence of citrus green mold [20]. Application of the biocontrol yeast *Pichia geleiformis* has been shown to enhance disease resistance in citrus fruit and upregulate the expression of transcription factors, including *CsMYB13*, *CsWRKY33*, *CsWRKY65*, *CsWRKY70*, and *CsERF098* [21]. Collectively, these findings indicate that transcription factors serve as central regulators of fruit disease resistance pathways and are often targeted or influenced by microbial signals.

However, the molecular mechanism through which yeast-secreted protein regulate host transcriptional networks remains unexplored. In light of the capacity of the yeast-secreted protein PgSCP to confer resistance against green mold in citrus fruits, this research initiates an investigation into the molecular pathways through which PgSCP enhances disease resistance. The research aims to screen and identify transcription factors that interact with PgSCP during the resistance response and to validate these interactions to clarify the functional role of PgSCP in enhancing disease resistance.

## Results

### PgSCP enhances disease resistance in citrus fruit

There was no apparent difference in colony diameter or growth morphology between the PgSCP-treated and PBS control plates, indicating that PgSCP does not directly inhibit *Penicillium digitatum* growth and thus lacks intrinsic antifungal activity (Supplementary Fig. 1). We examined the cell death response observed on the 10th day following transient overexpression of *PgSCP* in *N. benthamiana* leaves (Supplementary Fig. 2b). In order to investigate the function of PgSCP in the defense response of citrus fruit, we examined the activation of defense-related genes in citrus fruit that overexpress *PgSCP*. The transient overexpression of *PgSCP* in citrus fruit results in the significant upregulation of numerous genes associated with disease resistance, accompanied by a marked increase in disease resistance-related compounds (Supplementary Fig. 2). These findings suggest that the transient overexpression of *PgSCP* substantially enhances the disease resistance mechanisms in citrus fruit. And, PgSCP lacks transcriptional activation activity (Supplementary Fig. 2a). Therefore, the enhanced disease resistance observed in citrus fruit is likely attributed to the activation of disease resistance rather than direct antifungal activity.

### Characterization of PgSCP

**Figure 1:**
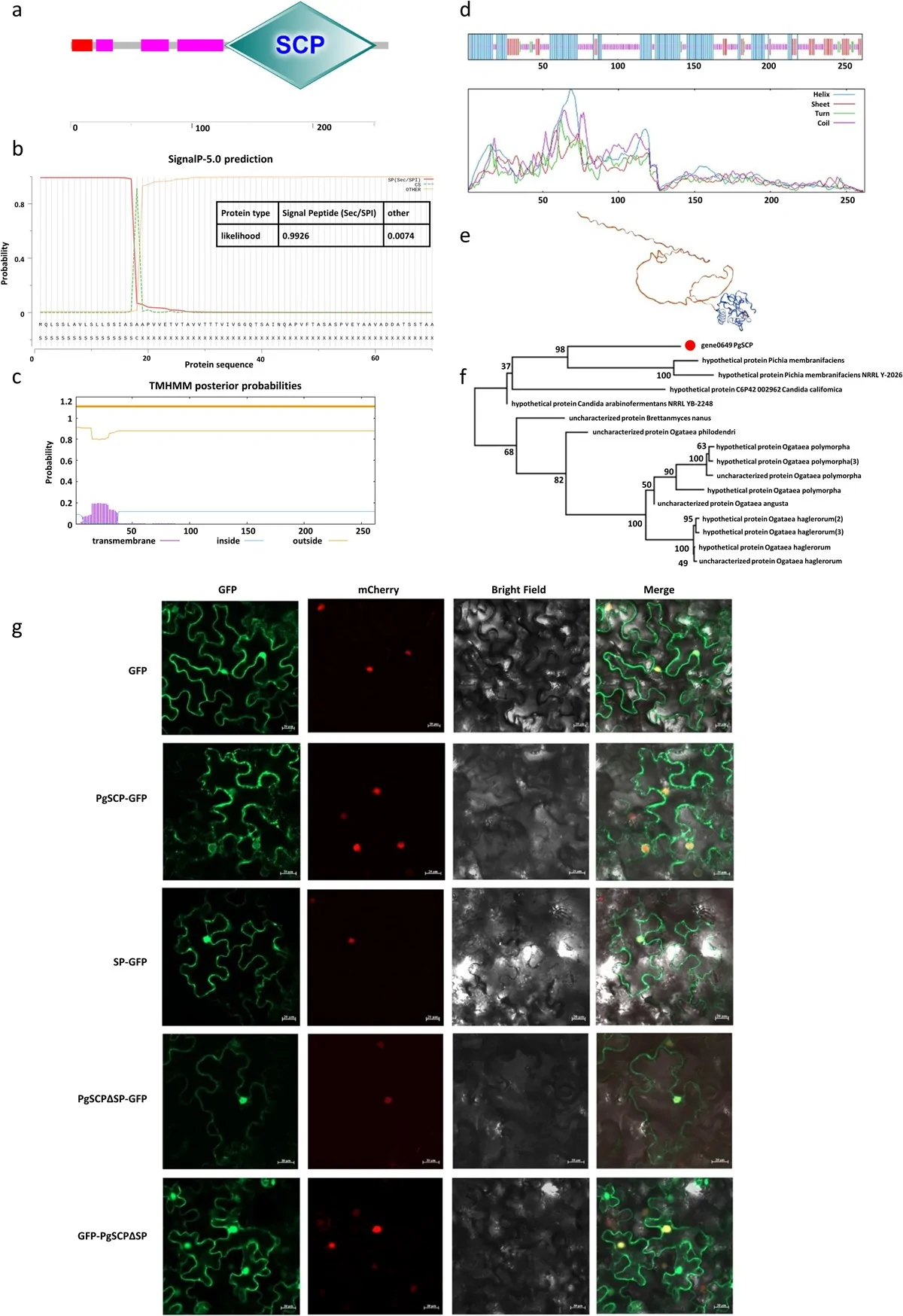
Characteristics of PgSCP. (a–c) Analysis of PgSCP conserved domains, signal peptides, and transmembrane domains. (d, e) Secondary and tertiary structure predictions of PgSCP. (f) Phylogenetic analysis of PgSCP. (g) Subcellular localization of PgSCP. Green fluorescence of merged images displayed the subcellular localization of PgSCP-GFP (complete sequence), SP-GFP (signal peptide sequence), and PgSCPΔsp-GFP and GFP-PgSCPΔsp (sequence without signal peptide) fusion protein. Bars, 20 μm.

The coding sequence (CDS) length of PgSCP is 789 bp, and the predicted molecular weight of the PgSCP protein is 26.89 kDa, comprising a total of 262 amino acids, which includes 6 cysteine residues (Cys, 2.29%). The protein is relatively enriched in several amino acids, notably alanine (Ala, 15.65%), aspartic acid (Asp, 6.11%), glutamic acid (Glu, 3.82%), glycine (Gly, 6.49%), and isoleucine (Ile, 2.67%). The PgSCP protein domain encompasses both a signal peptide and an SCP domain (Fig. 1a). The signal peptide prediction indicates that the N-terminal signal peptide of PgSCP consists of 18 amino acids, and PgSCP lacks a transmembrane domain, suggesting that it is located outside the yeast cell membrane (Fig. 1b and c). Consequently, it is hypothesized that PgSCP may be secreted from yeast cells into the extracellular environment and subsequently enter plant cells to perform its function. Hydrophilicity analysis yields a value of −0.011, suggesting that the protein exhibits hydrophilic characteristics. The computed instability index for the protein is 30.40, which is below the threshold of 50, indicating that the protein is stable. The predicted isoelectric point of PgSCP is 4.00. The secondary structure of the PgSCP protein is composed of random coils, α-helices, extended chains, and β-folds (Fig. 1d). Of these structural elements, random coils constitute the largest proportion at 41.42%, followed by α-helices at 40.46%. Extended chains and β-folds comprise 14.89% and 3.44% of the structure, respectively. The SCP domain represents the most significant component of the tertiary structure of PgSCP (Fig. 1e). Analysis of phylogenetic reveals that PgSCP shares a significant sequence similarity with a putative protein of unknown function (Fig. 1f). The construct pCAMBIA2300-GFP demonstrates green fluorescence ubiquitously within tobacco cells. Additionally, the fusion proteins PgSCP-GFP, SP-GFP, PgSCPΔSP-GFP, and GFP-PgSCPΔSP are all detectable with green fluorescence in both the nucleus and cytoplasm (Fig. 1g).

### Identification of citrus fruit proteins interacting with PgSCP

**Figure 2:**
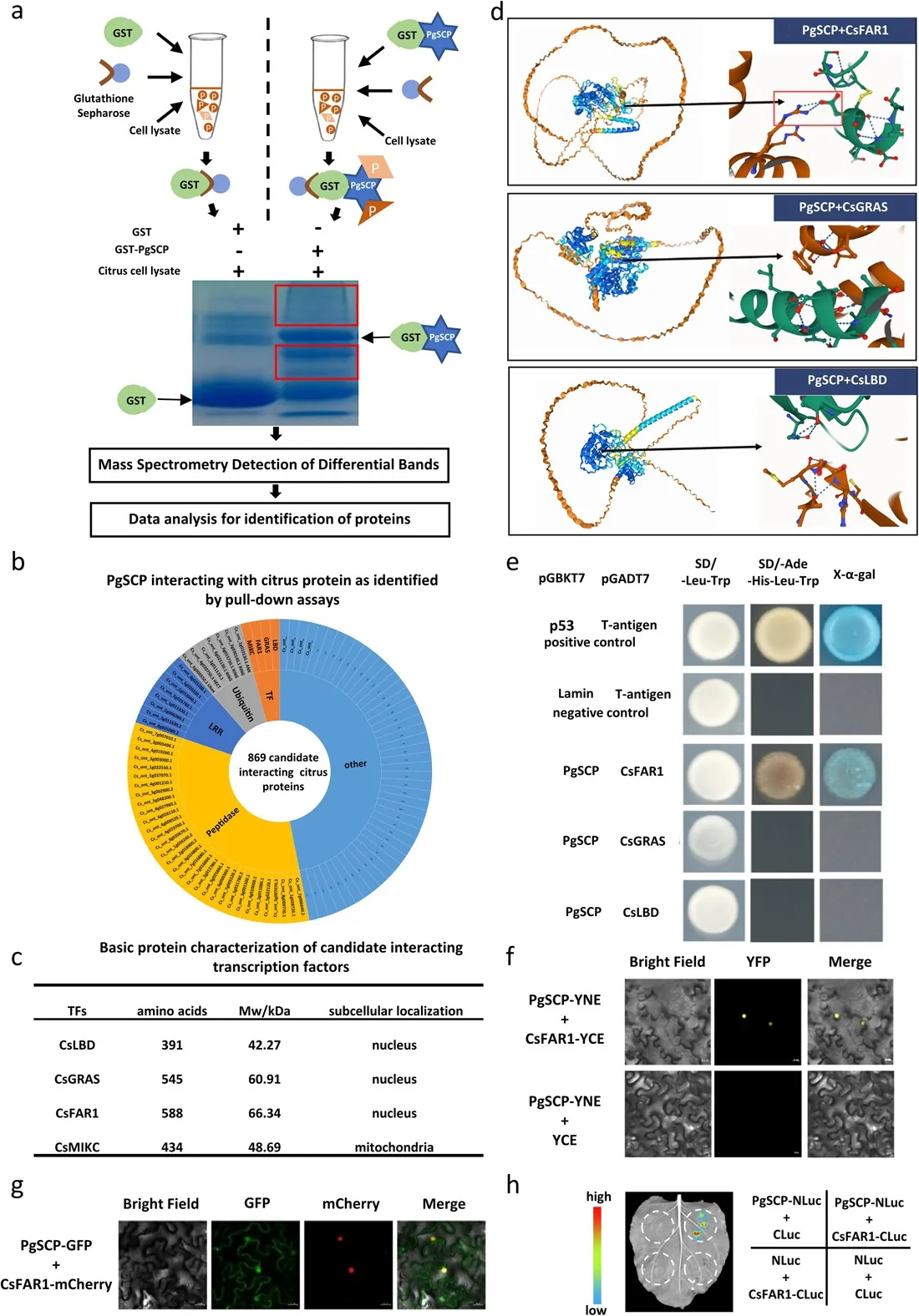
PgSCP interacted with citrus transcription factor CsFAR1. (a) Flowchart of the GST-pull down screening process for interaction proteins of PgSCP. (b) Identification of citrus proteins interacting with PgSCP. (c) Basic analysis of citrus transcription factors interacting with PgSCP. (d) Prediction of interactions between PgSCP and citrus transcription factors using AlphaFold3. (e) Verification of the interaction between PgSCP and CsFAR1 using Y2H. (f) Validation of the interaction between PgSCP and CsFAR1 using BiFC. (g) Subcellular co-localization analysis of PgSCP and CsFAR1. (h) Validation of the interaction between PgSCP and CsFAR1 using LCI.

As illustrated in Fig. 2a, the application of GST-PgSCP for the capture of citrus fruit proteins was confirmed through Coomassie Brilliant Blue staining. Concurrently, the results of protein electrophoresis revealed distinct variations in the protein bands between the PgSCP lane and control of the GST lane. This observation implies that PgSCP successfully captured the interacting citrus proteins. Through the identification of differential bands between GST-PgSCP and GST pull-down assays, 3141 peptides were analyzed, resulting in the identification of 869 candidate interactive proteins from citrus fruit (Supplementary Tables 1 and 2). The identification details, including protein IDs, conserved domains, homologous proteins, and predicted subcellular localizations, are presented in Supplementary Table 3. Initial investigations demonstrated a notable upregulation of various transcription factor families in the disease resistance response triggered by *P. galeiformis* and the yeast secretory protein PgSCP [21]. This suggests that transcription factors are crucial in the PgSCP-induced disease resistance mechanism. Subsequent analysis of potential interacting proteins identified four transcription factors (Fig. 2b).

The Cs_ont_6g011660.1 (CsFAR1) possesses the FAR1 conserved domain and has been identified as a transcription factor within the FAR1 family, with its subcellular localization predicted to be in the nucleus (Fig. 2c). The Cs_ont_7g012520.1 (CsMIKC) contains two conserved domains, namely MADS and K-box, and is classified as a member of the MIKC_MADS family, specifically of the AGL11 type, with its subcellular localization predicted to be in the mitochondria. The Cs_ont_3g011170.1 (CsLBD) is a transcription factor belonging to the LBD family, characterized by the presence of a conserved LOB domain. Its amino acid sequence includes a signal peptide, and its subcellular localization is predicted to be within the nucleus. Similarly, Cs_ont_4g020090.1 (CsGRAS) is a transcription factor of the GRAS family, featuring a conserved GRAS domain, with predictions also indicating its nuclear localization. Our research has indicated that PgSCP may operate within both the nucleus and cytoplasm of plant cells. Direct interactions between proteins within a cell typically suggest their colocalization within the same cellular compartment. Consequently, AlphaFold3 was employed to predict potential interactions between PgSCP and the proteins CsFAR1, CsLBD, and CsGRAS. The findings suggested a possible interaction between PgSCP and CsFAR1, while indicating that PgSCP is unlikely to interact with the transcription factors CsLBD and CsGRAS (Fig. 2d). The Y2H findings, presented in Fig. 2e, demonstrate that PgSCP interacts with CsFAR1 within the yeast cells. As demonstrated in Fig. 2f, fluorescence was observed in the nucleus of tobacco leaf epidermal cells co-transformed with PgSCP-YNE and CsFAR1-YCE. The luciferase complementation imaging (LCI) assay revealed a distinct luminescence signal upon the co-expression of *PgSCP* and *CsFAR1*, suggesting a specific interaction within plant cells (Fig. 2h). Furthermore, subcellular localization analysis demonstrated that PgSCP and CsFAR1 co-localize within the nucleus, thereby reinforcing the physiological significance of their interaction (Fig. 2g). These results indicate that PgSCP interacts with CsFAR1.

### The effect of PgSCP collaborates with CsFAR1 on the resistance to citrus green mold

**Figure 3:**
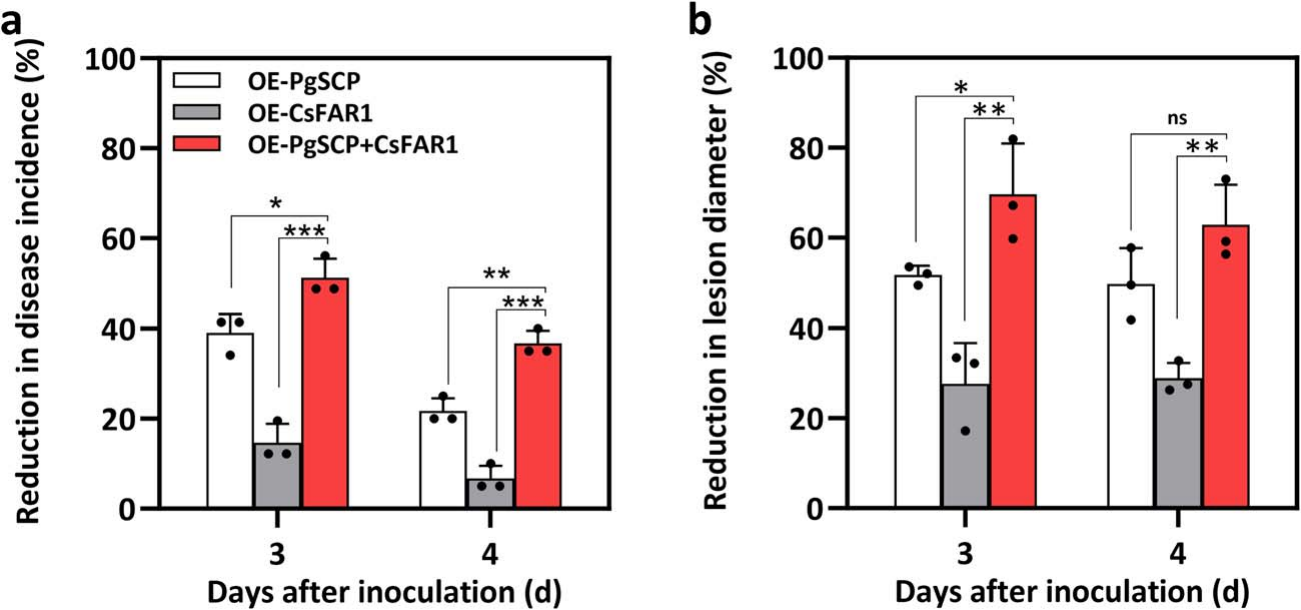
Effect of transient overexpression of *CsFAR1* in combination with *PgSCP*, respectively, on green mold resistance in citrus fruit. (a, b) Percentage reduction in disease incidence and lesion diameter compared to the empty vector control. Values represent means ± SD; statistical significance determined by LSD test (^*^P < 0.05, ^**^P < 0.01, ^***^P < 0.001, ns, *P* > 0.05).

The CsFAR1-GFP signal is exclusively observed within the cell nucleus (Supplementary Fig. 3a), indicating that the CsFAR1-GFP protein is specifically localized to the nucleus. As illustrated in Supplementary Fig. 3b, the LUC/REN ratios for both the positive controls and CsFAR1 were significantly elevated compared to the negative control, suggesting that CsFAR1 substantially enhances reporter gene expression. Further investigation into the effect of PgSCP in collaboration with CsFAR1 on the inhibition of green mold disease in citrus fruit was conducted. Compared to the control group, transient overexpression of *PgSCP* and *CsFAR1* induced a relative reduction in the incidence of green mold by 39.02% and 14.63%, respectively, at third day after inoculation. Whereas the combined overexpression of both genes during days 3–4 was significantly more effective in reducing disease incidence than individual treatments (Fig. 3). This suggests that the protein interactions between CsFAR1 and PgSCP, may augment the regulatory efficacy of the yeast secretory protein PgSCP in mitigating green mold in citrus fruit to a certain degree.

### The regulatory effect of CsFAR1 on disease-resistant genes in citrus fruit

**Figure 4:**
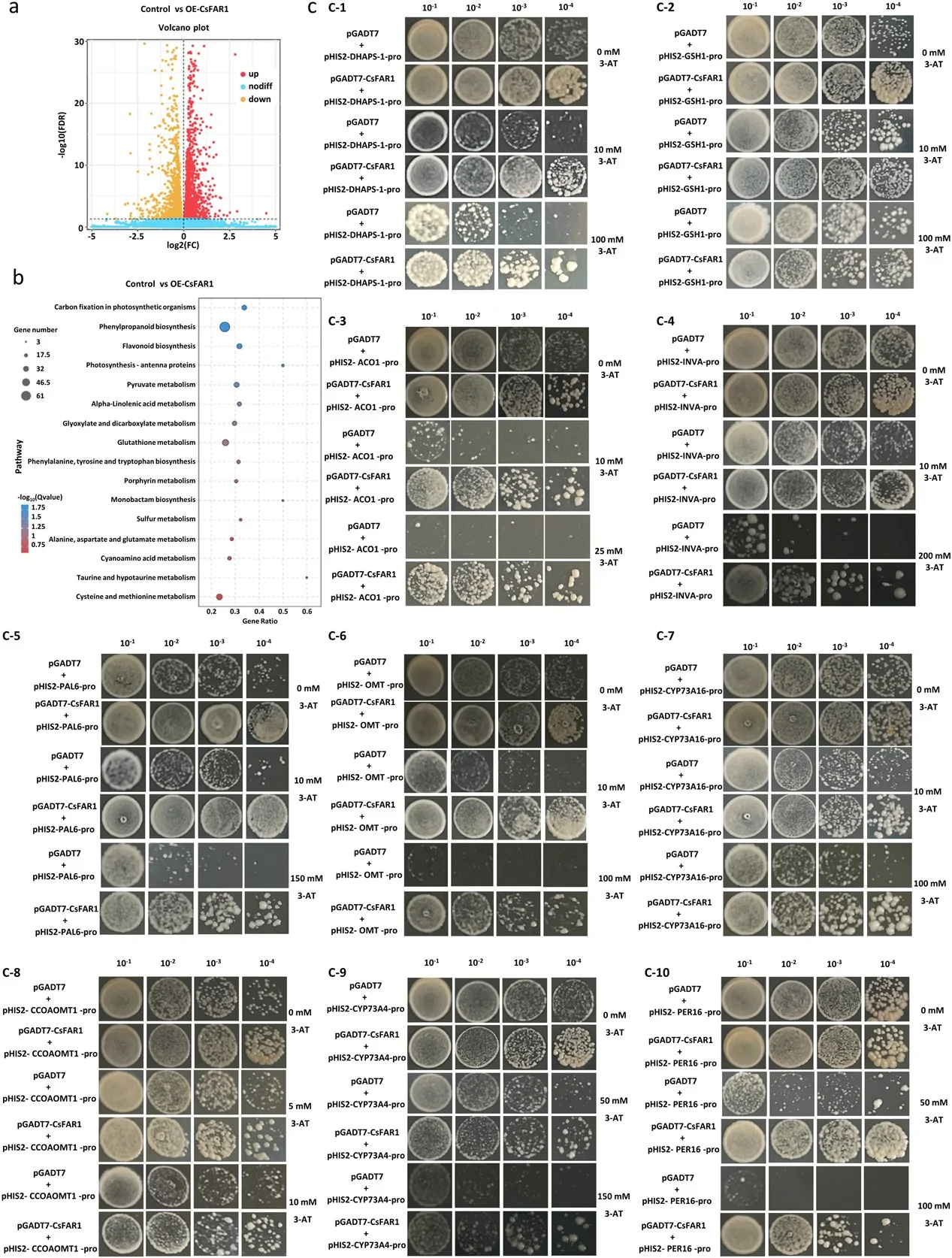
CsFAR1 combines with disease resistance-related target genes in citrus fruit. (a) Volcano plot of differentially expressed genes after transient overexpression of *CsFAR1*. (b) KEGG enrichment analysis of differentially expressed genes. (c) Y1H experiment analysis of CsFAR1 binds to promoter of *DHAPS-1* (c-1), *GSH1* (c-2), *ACO1* (c-3), *INVA* (c-4), *PAL6* (c-5), *OMT* (c-6), *CYP73A16* (c-7), *CCOAOMT1* (c-8), *CYP73A4* (c-9), and *PER16* (c-10).

Following the transient overexpression of the transcription factor *CsFAR1*, a total of 2872 genes were significantly differentially expressed, with 1525 genes being up-regulated and 1347 genes down-regulated (Fig. 4a). KEGG pathway enrichment analysis revealed several significantly enriched pathways associated with disease resistance, such as the phenylalanine, tyrosine, and tryptophan biosynthesis pathway (KEGG: 00400), cysteine and methionine metabolism pathway (KEGG: 00270), and phenylpropanoid biosynthesis (KEGG: 00940) (Fig. 4b). To further explore the downstream target genes regulated by CsFAR1, JASPAR was utilized to analyze potential promoters of downstream target genes that CsFAR1 may bind to, specifically focusing on differentially expressed genes within disease resistance-related pathways (Table 1).

**Table 1:** Jaspar analyzes the downstream target gene binding sites of CsFAR1.

Pathway	Gene ID	Start	End	Predicted sequence
Phenylalanine, tyrosine, and tryptophan biosynthesis	Cs_ont_4g003990 (*ASP3*)	1002	1010	TCACCCGCC
		33	41	CCACGCTCC
		248	256	ACCCGCGCG
		999	1013	CCGTCACCCGCCGCG
		30	44	AACCCACGCTCCAGC
	Cs_ont_2g014170 (*ASB1*)	1447	1455	TCACGTGCA
	Cs_ont_3g011210 (*DHAPS-1*)	1830	1838	CCACGTGCT
	Cs_ont_2g006920 (*TSB*)	1248	1256	TCACGCGGG
	Cs_ont_4g013970 (*At5g53970*)	663	671	GCACGCGCG
		1855	1863	CCACACGCT
		114	122	ACACGCACT
		1852	1866	ACACCACACGCTGCA
		660	674	TACGCACGCGCGCGC
		661	669	ACGCGCGCG
		160	168	ACACGTGCA
	Cs_ont_4g008300 (*ASP3*)	1692	1700	TCAGGCGCT
		1689	1703	GATTCAGGCGCTATT
Cysteine and methionine metabolism	Cs_ont_5g006290 (*METK1*)	919	928	GAACGCGCT
	Cs_ont_4g003990 (*ASP3*)	1002	1010	TCACCCGCC
		33	41	CCACGCTCC
		30	44	AACCCACGCTCCAGC
		248	256	ACCCGCGCG
		999	1013	CCGTCACCCGCCGCG
	Cs_ont_5g042040 (*Ldha*)	876	884	GCACGCGCT
		876	887	CGCGCACGCGCTTGT
		873	881	ACAAGCGCG
	Cs_ont_5g047950 (*GSH1*)	930	938	ACACGCGAG
	Cs_ont_2g001220 (*ACO*)	582	590	ACACGCACT
		714	722	ACACGCACT
		1909	1923	ATTTCACACGCAGAC
		777	785	TCACGCGGA
		580	588	TCACACGCA
		712	720	TCACACGCA
		1912	1920	TCACACGCA
		579	593	GTCACACGCACTAGC
		711	725	GTCACACGCACTAGC
	Cs_ont_2g018300 (*ACO1*)	107	115	TCACGCGTG
		110	118	TCACACGCG
		108	116	ACACGCGTG
		105	119	ATCACACGCGTGATT
		1678	1689	GCATGCGCC
	Cs_ont_5g003370 (*CAS3*)	1343	1351	GCACGCGCG
		1845	1853	CCACGCACC
		1340	1354	GGCGCACGCGCGTAT
		1340	1348	ATACGCGCG
		1842	1856	CCACCACGCACCTTC
		1337	1351	TACATACGCGCGTGC

(Continued)

To further validate the interaction between CsFAR1 and the promoters of downstream target genes associated with disease resistance, a yeast one-hybrid (Y1H) assay was performed. In this experiment, the promoters were co-transformed with pGADT7 into SD/-His/-Leu/-Trp medium, serving as a control. The findings revealed that the control group either failed to grow or exhibited poor growth on SD/-His/-Leu/-Trp medium supplemented with a specific concentration of 3-amino-1,2,4-triazole (3-AT). Conversely, CsFAR1 demonstrated robust growth following co-transfection with the promoters, suggesting that CsFAR1 can effectively bind to the promoters of the *DHAPS-1*, *GSH1*, *ACO1*, *INVA*, *PAL6*, *OMT*, *CYP73A16*, *CCOAOMT1*, *CYP73A4*, and *PER16* genes (Fig. 4c).

**Figure 5:**
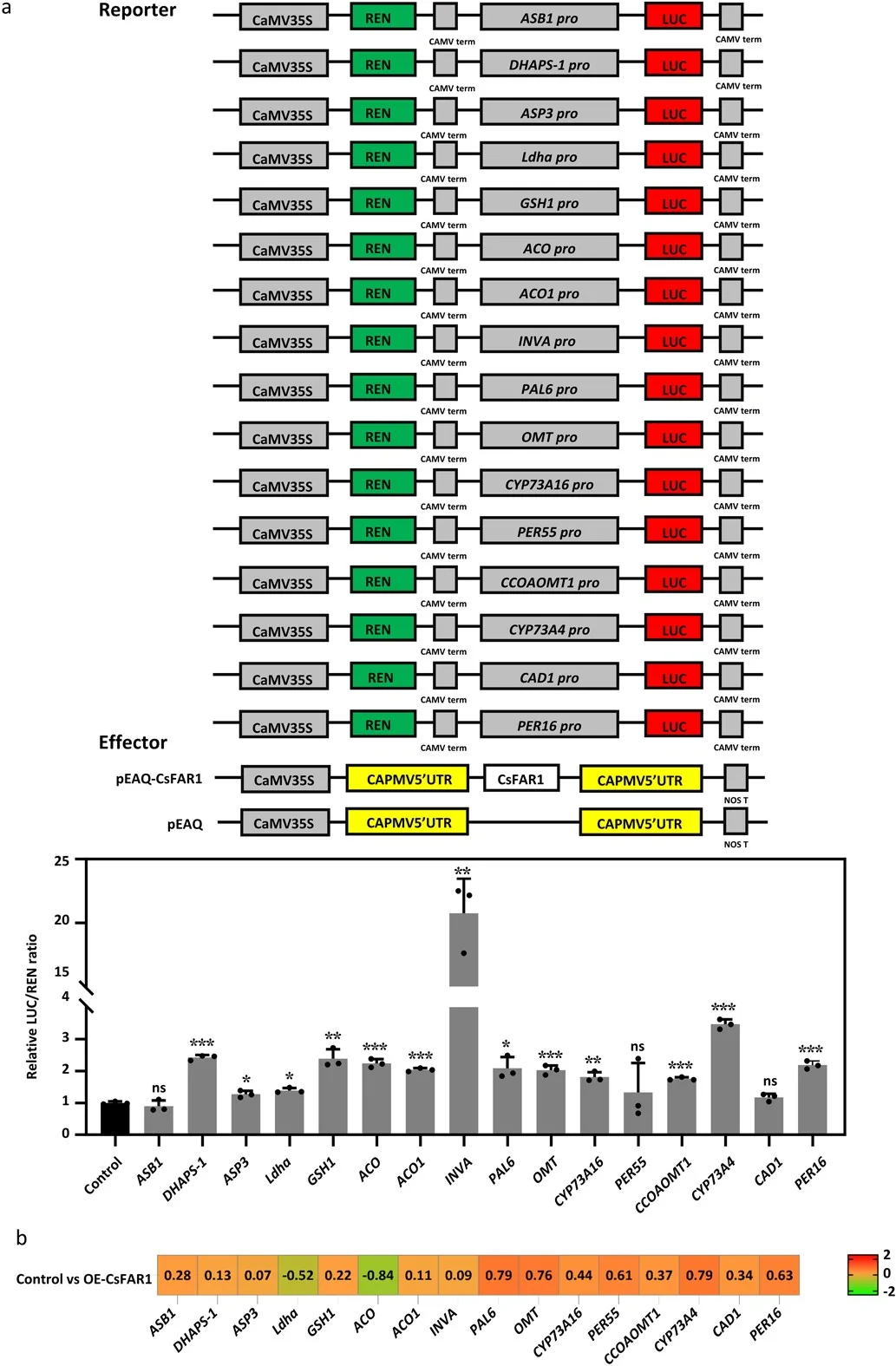
CsFAR1 activates the expression of target genes associated with disease resistance in citrus fruit. (a) The regulatory effect of CsFAR1 on the activity of promoters of disease resistance-related target genes. Values represent means ± SD; Statistical significance was assessed using an independent samples t-test to compare the experimental group and the control group (^*^P < 0.05, ^**^P < 0.01, ^***^P < 0.001, ns, *P* > 0.05). (b) After transient overexpression of *CsFAR1*, analyze the regulatory effect of CsFAR1 on disease resistance-related target genes.

As illustrated in Fig. 5, the LUC/REN ratio in tobacco leaves co-transfected with pEAQ-CsFAR1 and the promoters of *DHAPS-1*, *GSH1*, *ACO1*, *INVA*, *PAL6*, *OMT*, *CYP73A16*, *CCOAOMT1*, *CYP73A4*, and *PER16* was significantly elevated compared to the control group, which involved the co-transfection of pEAQ and the Reporter. These results indicate that CsFAR1 can activate the promoters of these genes. Consistent with this observation, transient overexpression of *CsFAR1* in citrus fruit led to marked upregulation of the expression of *DHAPS-1*, *GSH1*, *ACO1*, *INVA*, *PAL6*, *OMT*, *CYP73A16*, *CCOAOMT1*, *CYP73A4*, and *PER16*.

**Table 1:** Continued.

Pathway	Gene ID	Start	End	Predicted sequence
	Cs_ont_4g013970 (*At5g53970*)	663	671	GCACGCGCG
		1855	1863	CCACACGCT
		114	122	ACACGCACT
		1852	1866	ACACCACACGCTGCA
		660	674	TACGCACGCGCGCGC
		661	669	ACGCGCGCG
		160	168	ACACGTGCA
	Cs_ont_8g027150 (*BCAT2*)	899	907	TCACGCACC
		896	910	AACTCACGCACCCAT
		1417	1425	GCACGAGCC
	Cs_ont_4g008300 (*ASP3*)	1692	1700	TCAGGCGCT
		1689	1703	GATTCAGGCGCTATT
	Cs_ont_5g025860 (*AKHSDH1*)	1204	1212	ACACGCACT
		1206	1214	TCACACGCA
		1201	1215	GTCACACGCACTAGC
	Cs_ont_2g025370 (*AK2*)	1363	1371	CCACGCGCA
		1360	1374	AGTCCACGCGCACAC
		1641	1649	TTACGCGTC
	Cs_ont_9g008220 (*MD1*)	1739	1747	CCACGCGCA
		1736	1750	CACCCACGCGCACTC
		764	772	CCACGCTCT
		1731	1739	GCACGCACC
Phenylpropanoid biosynthesis	Cs_ont_7g006400 (*PAL*)	1727	1735	ACACGCGGC
	Cs_ont_5g030190 (*OMT*)	1559	1567	ACACGCTCT
		259	267	ACACTCGCT
		1556	1570	AAAACACGCTCTTCT
		1309	1323	ATTTGACCCGCTTCC
	Cs_ont_6g020620 (*PAL*)	1158	1166	CCACGTGCA
		1933	1941	TCACGCCCA
		1930	1944	CATTCACGCCCATTC
		1155	1169	AATCCACGTGCAAAC
	Cs_ont_1g006760 (*CYP73A16*)	1900	1908	CCACGCACA
		1482	1490	GCACGTGCT
	Cs_ont_2g024310 (*PER55*)	1584	1592	CCACGCGCT
		15 811	1595	TAGCCACGCGCTACT
		151	159	CCACGCACA
		222	230	CCACGCACA
	Cs_ont_8g024920 (*CCOAOMT1*)	640	648	TCACGCACC
		637	651	AAATCACGCACCATC
		943	951	TCCCGCGCG
		1032	1040	TCACGCTCA
		1800	1808	CCACGCTCA
	Cs_ont_4g024900 (*CYP73A4*)	296	304	ACACGTGCT
		293	307	AAAACACGTGCTTCA
		1391	1405	ATTGCACGCGTTTAA
		1394	1402	GCACGCGTT
	Cs_ont_1g011170 (*CCR1*)	522	530	ACACGTGCT
		519	533	AAAACACGTGCTTCC
	Cs_ont_2g034240 (*POD*)	1038	1046	CCACACGCC
		1035	1049	AATCCACACGCCACT
	Cs_ont_8g005310 (*PAL1*)	758	766	TCACGTGCG
		755	769	TCCTCACGTGCGTGC

(Continued)

**Table 1:** Continued.

Pathway	Gene ID	Start	End	Predicted sequence
	Cs_ont_8g025370 (*CAD1*)	874	882	TGACGCGCC
		871	885	TGCTGACGCGCCACA
	Cs_ont_4g025760 (*PER16*)	614	622	CCACGCGGT
		219	227	ACACTCGCT
		315	323	ACACACGCT
	Cs_ont_9g016320 (*COMT1*)	656	664	CCACGCGTT
		653	667	GATCCACGCGTTGCA
	Cs_ont_4g011080 (*UGT72E1*)	1604	1612	ACACACGCT
		1613	1621	TCACGCGTA
		1545	1553	TCACGTGCA
		1542	1556	AATTCACGTGCATTG
		1601	1615	GTAACACACGCTGCC
	Cs_ont_2g034280 (*POD*)	1951	1959	ATACGCGCC
		1948	1962	TATATACGCGCCTCT
	Cs_ont_7g021630 (*BGLU40*)	747	755	CCACGTGCA
	Cs_ont_4g012710 (*BGLU44*)	1157	1165	CAACGCGCG
		1155	1163	ACGCGCGCT
		1152	1166	TCAACGCGCGCTGTC
	Cs_ont_2g010710 (*BGLU40*)	1925	1933	TCACACGCC
		1922	1936	ATATCACACGCCTAT
	Cs_ont_5g016560 (*COMT1*)	324	332	ACCCGCGCT

### PgSCP induces disease resistance in citrus fruit via CsFAR1

**Figure 6:**
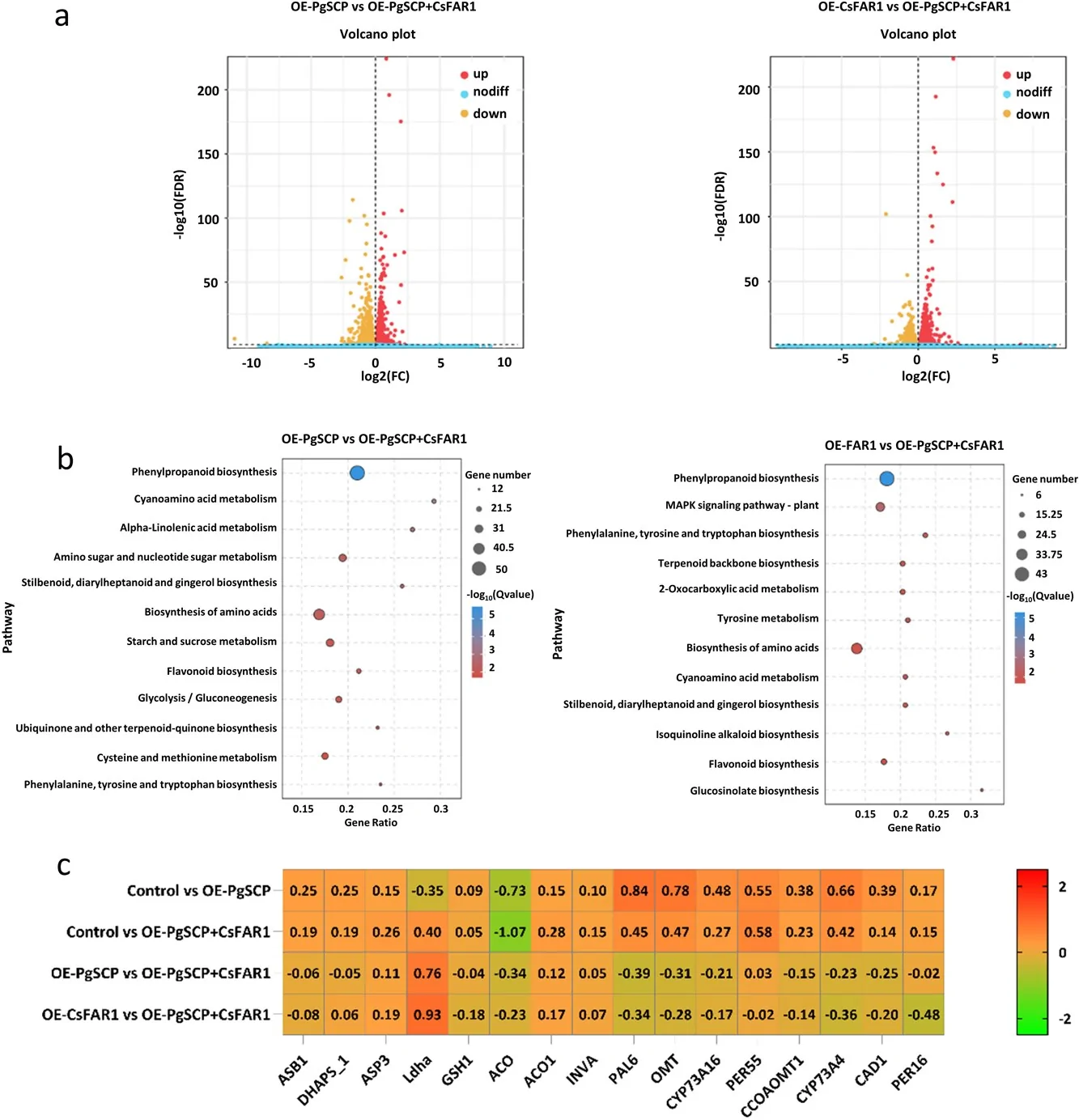
PgSCP induces disease resistance in citrus fruit via the CsFAR1. (a) Volcano plot of differentially expressed genes between transiently overexpressed *PgSCP* and co-transiently overexpressed *CsFAR1* and *PgSCP*, as well as between transiently overexpressed *CsFAR1* and co-transiently overexpressed *CsFAR1* and *PgSCP*. (b) KEGG enrichment analysis. (c) Analysis of the expression levels of disease resistance-related target genes after transient overexpression of *PgSCP* and co-transient overexpression of *CsFAR1* and *PgSCP*.

Upon co-transient overexpression of *PgSCP* and *CsFAR1*, a total of 798 genes were significantly upregulated, and 761 genes were significantly downregulated compared with *PgSCP* transient overexpression alone. Additionally, compared with *CsFAR1* transient overexpression alone, co-transient overexpression of *PgSCP* and *CsFAR1* resulted in 747 genes being significantly upregulated and 610 genes being significantly downregulated (Fig. 6a). KEGG pathway enrichment analysis revealed that the differentially expressed genes were significantly enriched in several pathways associated with disease resistance, including phenylpropanoid biosynthesis (ko00940), phenylalanine, tyrosine, and tryptophan biosynthesis (ko00400), flavonoid biosynthesis (ko00941), and cysteine and methionine metabolism (ko00270) (Fig. 6b). As illustrated in Fig. 6c, transient overexpression of *PgSCP* resulted in a significant upregulation of several CsFAR1 target genes, including *PAL6*, *OMT*, and *CYP73A16*, while the expression levels of *Ldha* and *ACO* were notably downregulated. Subsequent analyses demonstrated that co-transient overexpression of *PgSCP* and *CsFAR1* led to a pronounced upregulation of disease resistance-related genes such as *ASP3*, *Ldha*, *ACO1*, and *INVA*, compared to the individual transient overexpression of either *PgSCP* or *CsFAR1*. These findings indicate a synergistic interaction between PgSCP and CsFAR1 in modulating the disease resistance response in citrus fruit. This discovery further elucidates the role of the yeast-derived secretory protein PgSCP in influencing the activity of the citrus fruit transcription factor CsFAR1 within the context of disease resistance.

## Discussion

Microbial secreted proteins, especially effectors, play pivotal roles in either suppressing or triggering plant immunity [22–24]. A subset of secreted microbial proteins strongly triggers plant defense responses and could be developed into new biofungicides to boost plant disease resistance [25, 26]. However, the current understanding of the mechanisms by which secreted proteins from biocontrol yeasts elicit plant defense responses remains limited. Research has demonstrated that Secreted Cysteine-Rich Proteins (SCPs) from fungi activate plant defense mechanisms, including the induction of resistance genes and the generation of ROS, thereby conferring broad-spectrum resistance in plants. SCPs exhibit a high degree of conservation among numerous plant pathogenic fungal species. For instance, SCPs from *Valsa mali* are recognized by plant RLP proteins and trigger plant immunity, indicating the role of these proteins in plant defense [27]. Additionally, proteins such as BcSpl1 from *B. cinerea* and CS20EP from *Fusarium verticillioides* have been shown to enhance plant resistance to diseases. Furthermore, the Sm1 protein from the biocontrol fungus *Trichoderma* has the capability to induce systemic defense responses in cotton cotyledons [28, 29]. These findings suggest that cysteine-rich secreted proteins may act as important mediators of plant-microbe communication.

In this study, we investigated the function of a *P. galeiformis* Secreted Cysteine-rich Protein (PgSCP), a small secreted protein identified from the biocontrol yeast *P. galeiformis*. Our results demonstrate that PgSCP treatment is associated with delayed symptom development of green mold in citrus fruit, accompanied by the upregulation of defense-related genes and the accumulation of disease-resistance associated metabolites. Previous studies have indicated that the cysteine residues in microbial secreted proteins are often associated with the formation of intramolecular disulfide bonds, essential for protein stability and function [30]. Although prior research has established that cysteine-rich secreted proteins participate in plant defense responses, the relationship between their functional expression and the redox state of cysteine thiols remains unclear [31, 32]. To date, there have been no reports on yeast-secreted proteins containing SCPs regulating the defense responses of citrus fruit.

Secreted cysteine-rich proteins (SCPs) are widely distributed in fungi, oomycetes, and yeasts, playing crucial roles in plant-microbe interactions. Research shows fungal SCPs can function as apoplastic elicitors detected by plant receptors or as intracellular effectors targeting host immune systems. For instance, *Tilletia controversa* secretes TcSCP_9014, a protein that localizes to both the cytoplasm and nucleus, thereby modulating host immunity [32]. In *S. sclerotiorum*, an SCP is recognized by the plant receptor RLP30, triggering host immune responses [27]. More broadly, CAP superfamily proteins, which frequently contain cysteine residues, have been shown to manipulate plant immunity in *Fusarium oxysporum* [33]. These findings indicate that SCPs may employ a range of mechanisms, such as receptor interaction and intracellular targeting, to modulate host defense mechanisms.

Emerging evidence suggests that microbial secreted proteins access host cells via various pathways. Fungi and yeasts release extracellular vesicles (EVs) contain proteins, nucleic acids, and small metabolites. Schekman’s groundbreaking research, uncovered that yeast proteins are secreted through the ER-Golgi-vesicle pathway [34, 35]. Recent studies show that plant cells can absorb fungal EVs and their contents through clathrin-mediated endocytosis, enabling cross-kingdom transport of proteins and small RNAs [36, 37]. PgSCP, with its predicted N-terminal signal peptide, is likely secreted via the ER-Golgi pathway. It may be delivered into citrus cells by yeast-derived EVs to trigger defense responses.

Microbial secretory proteins engage with plants by exporting synthesized proteins outside the cell via various secretion systems and then transferring them into plant cells, where they target specific subcellular structures to execute their functions [38, 39]. Especially, the nuclear localization of microbial secretory proteins is crucial for their functionality, as certain microbial secretory proteins have the capacity to influence the transcriptional activity of transcription factors and other nuclear regulatory elements within plant cell nuclei. For example, the secretory protein PsAvh113 from *P. sojae* localizes in the nucleus and cytoplasm of plant cells and could bind to the soybean transcription factor GmDPB, reducing the transcription of the downstream target gene *GmCAT1*, thereby regulating plant immunity [40]. Additionally, the protein GcR8IP1 from *Golovinomyces cichoracearum* was secreted and transferred into the nucleus of *A. thaliana* cells. GcR8IP1 promotes the nuclear localization signal of RPW8.2, a powdery mildew resistance protein in *A. thaliana*, and interacts with RPW8.2 within the nucleus to mediate its functional effects [41]. Moreover, the secreted protein HaRxLL470 from the oomycete *Hyaloperonospora arabidopsidis* has been shown to interact with the plant transcription factor HY5. This interaction suppresses the plant defense against *H. arabidopsidis* infection. HY5 is pivotal in activating defense-related genes and positively regulates the plant resistance to pathogenic invasion [42]. In this study, the yeast secretory protein PgSCP was found to localize within the nucleus and cytoplasm of tobacco cells, suggesting a potential functional role for PgSCP in both compartments. This localization implies that PgSCP may similarly function within the nucleus and cytoplasm of citrus fruit. Furthermore, PgSCP could potentially interact with citrus transcription factors or other nuclear components, thereby influencing gene expression and modulating host defense response.

In order to further study how PgSCP interacts with biological factors-especially transcription factors in citrus fruit and thereby regulates the fruit defense response, we utilized GST-Pull Down technology to identify citrus proteins that potentially interact with PgSCP. Previous studies have established those certain microbial secretory proteins can directly interact with host transcription factors within the nuclei of host plant cells. Consistent with these observations, our findings confirmed that PgSCP localizes to both the nucleus and cytoplasm of plant cells. Moreover, transcription factors play a pivotal role in mediating plant defense responses. For instance, the transcription factor bHLH25 regulates two distinct defense pathways: lignin biosynthesis and antitoxin biosynthesis by modulating its oxidative/non-oxidative state while maintaining H₂O₂ levels, thereby effectively conferring resistance against a diverse array of pathogens [43, 44]. Therefore, we focused on analyzing citrus transcription factors that may interact with PgSCP.

Through a protein–protein interaction system, PgSCP was found to interact with the citrus fruit transcription factor CsFAR1. This is the first report of a biocontrol yeast-secreted protein interacting with host transcription factors to regulate the host disease resistance response. Analogously, the secreted protein VmSP1 from the pathogen *V. mali* has been identified as an inhibitor of plant immune responses. Further investigations revealed an interaction between VmSP1 and the apple transcription factor MdbHLH189. Transient overexpression of *MdbHLH189* was shown to upregulate the expression of defense-related genes in apple and promote callose deposition, thereby enhancing resistance against *V. mali* [45].

To further investigate the role of transcription factors in PgSCP-mediated induction of disease resistance in citrus fruit, this study found that the transient overexpression of *CsFAR1* positively regulate resistance to green mold citrus. This observation aligns with previous findings showing that several citrus transcription factors participate in disease resistance regulation. For instance, the overexpression of *CsAP2s* has been shown to enhance resistance to canker disease [46], while the co-overexpression of *CsAP2-09*, *CsWRKY25*, and *CsRBOH2* improves resistance to bacterial canker disease. Additionally, the overexpression of transcription factors such as *CsAP2L*, *CsERF1B*, and *CsERF098* has been demonstrated to enhance disease resistance in citrus fruit. Collectively, these findings underscore the pivotal roles of multiple citrus transcription factors in modulating disease resistance responses. Our results further suggest that the interaction between PgSCP and CsFAR1 enhances the efficacy of PgSCP in suppressing green mold, providing mechanistic insights into how biocontrol yeast proteins may reinforce host defense.

In the defense response of fruits, transcription factors typically regulate the disease resistance of fruits by suppressing or activating the expression of downstream genes, thereby controlling downstream metabolic pathways. For example, the transcription factors CsAP2L and the CsAP2–09-CsWRKY25-CsRBOH2 cascade enhance the disease resistance of citrus fruit against bacterial canker by regulating the expression levels of key genes involved in lignin biosynthesis and ROS metabolism pathways [47]. Our study reveals that CsFAR1 binds to the promoter regions of multiple defense-related genes, including *DHAPS-1*, *GSH1*, *ACO1*, *INVA*, *PAL6*, *OMT*, *CYP73A16*, *CCOAOMT1*, *CYP73A4*, and *PER16*, directly modulating their expression. These genes are implicated in pathogen recognition, signal transduction, and defense responses, indicating that CsFAR1 enhances host disease resistance via a multi-layered regulatory network.

**Figure 7:**
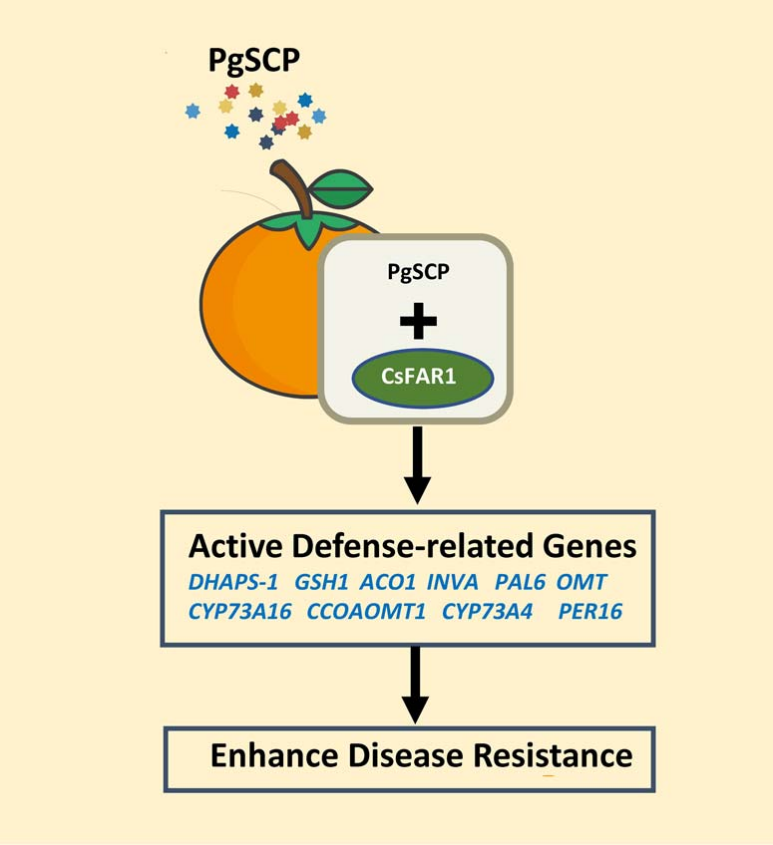
The schematic representation of yeast secreted protein PgSCP synergized with citrus transcription factor CsFAR1 regulating the resistance of citrus fruit to green mold.

In summary, this study demonstrates that the secreted protein PgSCP from the biocontrol yeast interacts with the citrus fruit transcription factor CsFRA1 to activate expression of disease-resistance-associated genes, including *DHAPS-1*, *GSH1*, *ACO1*, *INVA, PAL6*, *OMT*, *CYP73A16*, *CCOAOMT1*, *CYP73A4*, and *PER16*. This interaction enhances green mold resistance in citrus fruits by strengthening citrus defense resistance (Fig. 7). Notably, PgSCP functions as a microbe-associated molecular pattern (MAMP)-like elicitor, inducing host defense responses rather than exhibiting direct fungicidal activity. This mode of action reduces the risk of pathogen resistance and environmental contamination. Therefore, our findings provide a conceptual and technical foundation for developing sustainable, yeast-derived protein-based biocontrol products for postharvest citrus protection. These findings advance understanding of yeast-mediated defense activation and provide a mechanistic basis for developing strategies to improve citrus disease resistance.

## Materials and methods

### The effect of PgSCP on the growth of *P. digitatum*

To determine whether PgSCP exerts a direct inhibitory effect on *P. digitatum*, an Oxford cup assay was performed [48]. Briefly, 10 ml of 2% sterile agar was first poured into a Petri dish and allowed to solidify. A sterile Oxford cup was then gently placed at the center of the plate, followed by the addition of another 10 ml PDA medium on top. Subsequently, 20 μl 1 × 10^6^ spores ml^−1^  *P. digitatum* spore suspension was added into the well and allowed to absorb. Then, 200 μl of purified PgSCP protein solution (100 μmol l^−1^, glutathione-free) was added to the same well. PBS buffer was used as a negative control. Plates were placed at 4°C for 2 h to allow diffusion and then incubated at 25°C. The growth of *P. digitatum* colonies was monitored and photographed after incubation.

### The effect of PgSCP induction on the disease resistance of citrus fruit

Following the transient overexpression of the yeast secretory protein PgSCP in citrus fruit, peel samples were collected for transcriptomic and broad-target metabolomic analyses 2 days post-treatment. Each experimental condition was replicated three times. Transcriptomic sequencing was performed by Guangzhou Kidio Biotechnology Co., Ltd., utilizing the sweet orange genome (Citrus sinensis v3.0) as the reference genome. The RNA-seq data was deposited in NCBI SRA database under accession number SRR34942147. The broad-target metabolomic analysis was carried out by Wuhan Metviare Biotechnology Co., Ltd.

### Characterization of PgSCP protein

Using the online software SignalP 5.0 (https://services.healthtech.dtu.dk/services/SignalP-5.0/) to analyze the distribution of signal peptides in the first 60 amino acids of the PgSCP protein sequence, and utilizing TMHMM (https://services.healthtech.dtu.dk/services/TMHMM-2.0/) online software for transmembrane domain prediction of the PgSCP amino acid sequence. The online software Sequence Manipulation Suite was used to predict the molecular weight, amino acid composition, isoelectric point, instability index, and hydrophilicity of the PgSCP protein. ProtParam (http://web.expasy.org/protparam/) was employed to predict the molecular weight, theoretical isoelectric point, instability index, and hydrophilicity of the amino acid sequence. Subcellular localization prediction was conducted using the online software CELLO (http://cello.life.nctu.edu.tw/); nuclear localization signal prediction was carried out using cNLS Mapper (http://nls-mapper.iab.keio.ac.jp/cgi-bin/NLS_Mapper_form.cgi). The ProtParam online tool was used to predict the primary structure of PgSCP; the SOPMA online tool (https://npsa-prabi.ibcp.fr/cgi-bin/npsa_automat.pl?page=/NPSA/npsa_sopma.html) was used to predict the secondary structure of the protein. Amino acid sequence alignment to find homologous proteins of PgSCP was performed on the NCBI website (http://www.ncbi.nlm.nih.gov/); DNAMAN software was used for multiple sequence alignment of related proteins; and MEGA 6.0 software was used to construct phylogenetic trees.

The construction of PgSCP fusion GFP expression vectors, including GFP-PgSCP and PgSCPΔSP-GFP (lacking the signal peptide region), as well as GFP-PgSCPΔSP and the SP fusion GFP expression vector SP-GFP, was undertaken [49]. These constructs were co-infiltrated into the leaves of *N. benthamiana* using *Agrobacterium* containing a nuclear marker. After a 48-h incubation period, the results were observed and documented using a laser confocal microscope.

### Identification of proteins in citrus fruit that interact with PgSCP

#### Extraction of citrus fruit proteins

Proteins from citrus fruit were extracted using a plant Western and immunoprecipitation (IP) cell lysis buffer (Biyuntian, catalog number: P0043). Initially, one gram of frozen citrus peel was precisely weighed and ground into a fine powder with the aid of liquid nitrogen to achieve thorough homogenization. The resultant powder was then transferred into a 50 ml centrifuge tube, where 6 ml of lysis buffer, supplemented with protease inhibitors and phenylmethylsulfonyl fluoride (PMSF) at a final concentration of 1 mmol l^−1^, was added. Thoroughly homogenize the sample. Employ an ultrasonic cell disruptor to lyse the cells, operating at a maximum power of 1000 W and set to 20% power, while maintaining the sample on ice for a duration of 15 min. Upon achieving complete cell lysis, centrifuge the lysate at 4 °C at a speed of 12 000 rpm for 10 min to isolate the supernatant, which contains the citrus fruit protein. Quantify the protein concentration using a BCA assay kit. Subsequently, perform SDS-PAGE followed by Coomassie Brilliant Blue staining to visualize the protein bands.

#### GST-pull down assay coupled with Mass Spectrometry (GST-pull down/MS)

To identify citrus fruit proteins interacting with PgSCP, a pull-down assay was conducted [50, 51]. The purified GST-PgSCP protein and a GST-tagged protein serving as a negative control were combined with a citrus peel protein extract in 10 ml Eppendorf tubes, with an appropriate quantity of protease inhibitors and phenylmethylsulfonyl fluoride (PMSF) added. This mixture was incubated at 4 °C on a rotator for a duration of 3 h. Following incubation, the protein mixture was transferred to EP tubes containing glutathione resin and subjected to overnight incubation at 4 °C on a rotator. Subsequently, the mixture underwent centrifugation at 500 rpm for 2 min at 4 °C, after which the supernatant was discarded. The glutathione resin pellet was then washed with 1.5 ml of pre-cooled PBS, a process repeated five times to ensure the removal of unbound contaminants. Finally, the pellet was resuspended in 200 μl of 2 × SDS sample buffer, resulting in the pull-down product.

Utilize 20 μl of the pull-down product for SDS-PAGE analysis to ascertain the presence of GST-tagged proteins and GST-PgSCP proteins within the sample. Conduct mass spectrometry analysis on the differential bands observed in the PgSCP-GST pull-down lane. The mass spectrometry analysis of these differential bands was outsourced to Shanghai Zhongke New Life Biotechnology Co., Ltd.

#### Bioinformatics analysis of citrus fruit proteins interacting with PgSCP

The corresponding protein sequences were retrieved from the third-generation genomic database of sweet orange at Huazhong Agricultural University utilizing the designated protein identifiers. Subsequently, domain prediction of these proteins was conducted using the SMART online tool (https://smart.embl.de/). Furthermore, the online tool Plant-mPLoc (http://www.csbio.sjtu.edu.cn/bioinf/plant-multi/) was employed to predict the subcellular localization of the proteins. Additionally, a BLAST analysis of homologous proteins corresponding to the candidate interacting proteins was performed using the NCBI database. The identified proteins from citrus fruit were utilized to extract protein sequences using their respective protein IDs through Java programming. Subsequently, these protein sequences were employed for transcription factor prediction within the Plant Transcription Factor Database (PlantTFDB) (http://planttfdb.gao-lab.org/). Utilizing the AlphaFold3 model (https://alphafoldserver.com/welcome) to predict protein–protein interactions [52]. Then, fundamental protein characteristics of the candidate interacting transcription factors were analyzed using the online tool Expasy (https://web.expasy.org/compute_pi/).

### Verification of the interaction between PgSCP and citrus fruit transcription factors

#### Yeast two-hybrid (Y2H) assay

To investigate potential self-interaction of the PgSCP protein, yeast two-hybrid vectors pGBKT7-PgSCP and pGADT7-PgSCP were constructed. The PgSCP protein and its interacting transcription factors were cloned into the pGBKT7 and pGADT7 vectors, respectively, and subsequently transformed into Y2H Gold yeast competent cells [53]. The resulting colonies were stained with X-α-GAL to assess interactions between PgSCP and citrus transcription factors. Observations were documented through photographic evidence.

#### Bimolecular Fluorescence Complementation (BiFC) assay

The genes PgSCP and citrus transcription factors were cloned into nYFP and cYFP vectors, respectively, and subsequently transformed into *Agrobacterium tumefaciens* strain GV3101. Following the co-cultivation of the two *Agrobacterium* strains, the resultant mixture was infiltrated into *N. benthamiana* leaves [53]. Fluorescence was then examined using a laser scanning confocal microscope, and images were captured 2–3 days post-infiltration.

#### Luciferase complementation imaging (LCI) assay

Recombinant *Agrobacterium* strains harboring CsFAR1-NLuc and PgSCP-CLuc constructs were cultured and subsequently resuspended in infiltration buffer to an optical density at 600 nm of 0.5. The bacterial suspensions were mixed in equal proportions and infiltrated into the leaves of *N. benthamiana*. After 2 days, the leaves were treated with potassium luciferin and incubated in darkness for 15 min. Chemiluminescence was subsequently captured using an imaging detection system (Vilber, Fusion Solo6s).

### Subcellular co-localization of CsFAR1 and PgSCP

Recombinant *Agrobacterium tumefaciens* strains containing CsFAR1-mCherry, PgSCP-GFP, and P19 were cultured in antibiotic LB medium, centrifuged, and resuspended in infiltration buffer to an OD_600_ of 0.8. After incubating at 28 °C in the dark for 2–3 h, cultures were mixed and infiltrated into *N. benthamiana* leaves. After 48 h, leaf samples were analyzed using a laser scanning confocal microscope.

### Subcellular localization and transcriptional activation of CsFAR1

Develop expression vectors for CsFAR1 by fusing them with green fluorescent protein (GFP). Employ an *Agrobacterium*-mediated transient expression system to infiltrate the bacterial solution containing the positive clones, along with a nuclear marker, into tobacco leaves. Following a 48-h incubation period, utilize a laser confocal microscope to observe and document the infiltrated leaves through photography.

The interactive transcription factors CsFAR1 were cloned into the plant expression vector pEAQ-BD (pBD) via the Stu I restriction enzyme site and subsequently transformed into the *Agrobacterium* strain EHA105. A recombinant plasmid incorporating the CaMV35S promoter functioned as the effector, whereas a vector containing a TATA-box linked to the GAL4-LUC and REN dual reporter genes was utilized as the reporter. The effector and reporter constructs were co-transformed into tobacco leaves at a 9:1 ratio. Following a cultivation period of 2–3 days, pBD-VP16 and the pBD empty plasmid served as positive and negative controls, respectively. Leaf samples were collected using a puncher, and the activity ratio of firefly luciferase to Renilla luciferase was quantified utilizing a commercial assay kit. Each experimental condition was assessed with six biological replicates.

### The effect of PgSCP collaborating with citrus transcription factors on the resistance to green mold

The impact of co-expressing PgSCP with CsFAR1 on enhancing the resistance of citrus fruit to green mold was examined, utilizing *P. digitatum* as an indicator pathogen. The genes encoding *PgSCP* and *CsFAR1* were individually cloned into the plant dual expression vector pEAQ, and the resulting recombinant plasmids were subsequently introduced into *Agrobacterium* strain GV3101. The experimental design comprises treatment groups characterized by the co-expression of *PgSCP* with *CsFAR1*, the singular expression of *PgSCP*, the singular expression of *CsFAR1,* and Control group (empty vector). The reduction in disease incidence and lesion diameter of citrus fruit was expressed as a percentage compared to the control group, calculated using: (Control Value - Treatment Value) / Control Value × 100%.

### Transcriptomic analysis of the induction of disease resistance against green mold in citrus fruit mediated by PgSCP and CsFAR1

Samples from the co-transient overexpression of *PgSCP* and *CsFAR1*, the singular expression of *PgSCP*, the singular expression of *CsFAR1,* as well as the transient overexpression of an empty vector as control, were collected 2 days post-transfection for RNA-seq analysis. Each experimental condition was represented by three biological replicates. The RNA-seq analysis was performed by Guangzhou Kidio Biotechnology Co., Ltd.

### Prediction of transcription factor and downstream target gene binding sites

Using JASPAR (https://jaspar.elixir.no/) to predict transcription factor binding sites [54].

#### Yeast one-hybrid (Y1H) assay

The pHIS2 vector was ligated to a 100 bp fragment encompassing the target gene binding element, and the CsFAR1 gene was cloned into the AD vector. The experimental group comprises the pHIS2-bait and AD-CsFAR1 plasmids, whereas the self-activation control group includes the pHIS2-bait and empty AD plasmids. Both groups were co-transformed into Y187 chemically competent cells and cultured in SD/-Leu/-Trp liquid medium. Following the screening process, positive clones were propagated in SD/-Leu-Trp liquid medium. Subsequently, the bacterial suspension was subjected to a tenfold dilution prior to being applied onto SD/-His/-Leu/-Trp agar plates, which contain a gradient of 3-amino-1,2,4-triazole (3-AT). The concentration of 3-AT was determined based on the amplification characteristics of the self-activating colony group. The formation of colonies on the SD/-His/-Leu/-Trp plates serves as an indicator of the interaction between CsFAR1 and the target gene.

#### Dual-luciferase reporter (DLR) assay

The downstream target gene was cloned into the pGreenII 0800-LUC vector. The pEAQ vector functioned as a negative control, while pEAQ-CsFAR1 was employed as an effector. The promoter of the downstream target gene was utilized as the reporter gene [55]. Subsequently, the constructs were introduced into tobacco plants following a 2–3 h treatment with an osmotic agent. Measurements were conducted 48 h post-treatment. The transcriptional activation or inhibition capacity of CsFAR1 on the target genes is quantified by the LUC/REN ratio, with each experimental condition replicated a minimum of six times.

### Statistical analysis

All primers used in this study are listed in Supplementary Table 4. An independent-samples t-test was employed to compare the data using SPSS version 23.0 (SPSS Inc., Chicago, IL, USA). A *P*-value of less than 0.05 was considered statistically significant. Figures were generated using GraphPad Prism software.

## Supplementary Material

Web_Material_uhaf339

## Data Availability

All relevant data are available within the manuscript and its supplementary data.
